# Analysis of the prevalence and associated factors of overactive bladder in adult Korean men

**DOI:** 10.1371/journal.pone.0175641

**Published:** 2017-04-13

**Authors:** So Young Kim, Woojin Bang, Hyo Geun Choi

**Affiliations:** 1 Department of Otorhinolaryngology-Head & Neck Surgery, CHA Bundang Medical Center, CHA University, Seongnam, Korea; 2 Department of Urology, Hallym University Sacred Heart Hospital, Hallym University Sacred Heart Hospital, Anyang, Korea; 3 Department of Otorhinolaryngology-Head & Neck Surgery, Hallym University College of Medicine, Anyang, Korea; Cedars-Sinai Medical Center, UNITED STATES

## Abstract

Overactive bladder (OAB) is a prevalent condition characterized by lower urinary tract symptoms (LUTS). Age, education, income, marital status, sleep, and emotional problems have been associated with OAB; however, conflicting results exist. The present study was conducted to estimate the prevalence of OAB and comprehensively analyze its associated factors in a large cross-sectional, population-based study. The data of 94,554 participants aged 19–107 were analyzed from the Korean Community Health Survey (KCHS) of 2012. Data on marital status, physical activity, education level, occupation, body mass index (BMI), income level, sleep time, and stress level were retrieved for all enrolled participants. The overactive bladder symptom score (OABSS) was used to evaluate the presence and degree of OAB. Simple and multiple logistic regression analyses with complex sampling were used for the associations between various factors and the presence of OAB. Overall, OAB was present in approximately 2.9% of the participants. The prevalence of OAB increased with age and steeply increased after 60 years of age (adjusted odds ratio [AOR] for each 10 years = 1.70, 95% confidence interval [CI] = 1.61–1.80, P<0.001). The prevalence of OAB was lower in married than unmarried subjects (AOR = 0.59, 95% CI = 0.48–0.72, P<0.001). The prevalence of OAB was significantly different according to occupation Compared to manager, expert, specialist, clerk group, the prevalence of OAB was highest in unemployed group (AOR = 1.90, 95% CI = 1.55–2.32, P < 0.001). Being underweight was correlated with OAB (AOR = 1.29, 95% CI = 1.08–1.55, P = 0.018). Inadequate sleep showed a significant association with OAB (AOR = 1.13, 95% CI = 1.02–1.25 for ≤6 hours of sleep time and AOR = 1.53, 95% CI = 1.27–1.86 for ≥9 hours of sleep, P<0.001). Stress level showed a dose-dependent positive association with OAB [AOR (95% CI) = 3.91 (3.13–4.89) > 2.16 (1.88–2.48) > 1.39 (1.23–1.57) for severe stress > moderate stress > some stress, respectively, P<0.001]. A medical history of diabetes mellitus, hyperlipidemia, and/or cerebral stroke was significantly related to OAB. Approximately 2.9% of adult Korean men experienced OAB based on the OABSS. Unmarried status; occupation; being underweight; inadequate sleep; stress; and medical history of diabetes mellitus, hyperlipidemia, or cerebral stroke were significantly correlated with OAB.

## Introduction

Overactive bladder (OAB) is characterized by lower urinary tract symptoms (LUTS), including urgency and frequency of urination, associated with storage problems and nocturia [1]. OAB has received much attention because of its high prevalence and various impacts on life. Previous studies have reported a prevalence in men of approximately 10–20%, which is comparable to women [2–4]. However, depending on the study population and investigation methods, the prevalence of OAB was estimated to be as low as 6.0% in the adult population in China and as high as 45.9% in the African-American population [5, 6].

OAB is known to be detrimental to the overall quality of life because of its impact on physical, emotional, and social aspects [3, 7, 8]. OAB is related to the various psychological problems of sleep disturbance, low self-esteem, depression, and anxiety as well as urinary complaints and subsequent diminished sexual activity [9]. Approximately 50% of OAB patients complained of decreased physical activity because of OAB [10]. OAB led to decreased work productivity, with 2–3 times more absent days from work [11]. Unemployment was approximately 1.5 times higher in men with OAB than in normal controls [12]. Therefore, OAB is associated with a large economic burden [3, 7, 8].

The pathophysiologic mechanism of OAB has not been well established. Abnormal spontaneous motor activity of the detrusor muscle and the release of neurotransmitters by the urothelium have been suggested to trigger urgency [13]. Additionally, the urothelium of the bladder is presumed to play a key role in the mechanosensory transduction and causes of OAB [14]. Bladder function is also regulated by central mechanisms [15]. Thus, central neurologic problems, such as an ischemic cortical brain lesion, could induce OAB. Because of these diverse pathophysiologic mechanisms, various factors have been related to OAB. In addition to aging, socio-economic factors, such as education; economic level; marriage; and psychological factors, including lack of sleep, depression, and anxiety, were demonstrated as associations in previous studies [5, 16]. In addition, several medical disorders, including metabolic syndrome and stroke, failed to show an association with OAB [13, 17].

However, some conflicts exist related to the association between various factors and OAB. For instance, the relationship of OAB to obesity has been a controversial topic [18]. Most previous studies considered only some of these variables, and few studies concurrently analyzed numerous factors. Moreover, until recently, a consensus has not been available concerning the definition of OAB. Earlier studies reported that OAB was characterized by the prevalence of urinary incontinence and nocturia in a specific age group [19, 20], and OAB was investigated in the presence of urgency without verified survey items [2, 16]. Although a population-based study was conducted in Korean men, the study was conducted by telephone survey, with a response proportion as low as approximately 22.1% [16]. Therefore, we conducted a large population-based, cross-sectional survey to evaluate the prevalence and associated factors of OAB in Korean men. To our knowledge, a large population-based study based on the validated questionnaire of overactive bladder symptom score (OABSS) has not been conducted [21]. Additionally, our study involved multivariate analyses with numerous physical, psychological, and socio-economic factors.

## Materials and methods

### Study population and data collection

This study was approved by the Institutional Review Board of Korea Centers for Disease Control and Prevention (IRB No. 2012-07CON-01-2C). Written informed consent was obtained from all of the participants prior to the survey. All of the Korean Community Health Survey (KCHS) were conducted in accordance with the guidelines and regulations which provided by the Korea Centers for Disease Control and Prevention [22].

This study was a cross-sectional study using data from the KCHS. The data from the 2012 KCHS were analyzed. The data were collected by the Centers for Disease Control and Prevention of Korea. The survey gathered information through face-to-face, paper-assisted personal interviews between trained interviewers and respondents. The sample size for the KCHS was 900 subjects in each of 253 community units, including16 metropolitan cities and provinces. Detailed description including total population and sampling household and participants were reported in KCHS website [22]. The KCHS used a two-stage sampling process. The first stage selected a sample area (administrative district: tong/ban/ri) as a primary sample unit, which was selected according to the number of households in the area using a probability proportional to the sampling method. In the second stage, the number of households in the selected sample tong/ban/ri was identified to create a household directory. Sample households were selected using systematic sampling methods. This process was used to ensure that the sample units were representative of the entire population [23]. For the sample to be statistically representative of the population, the data collected from the survey were weighted by statisticians based on the sample design (S1 File) [24].

**Fig 1:**
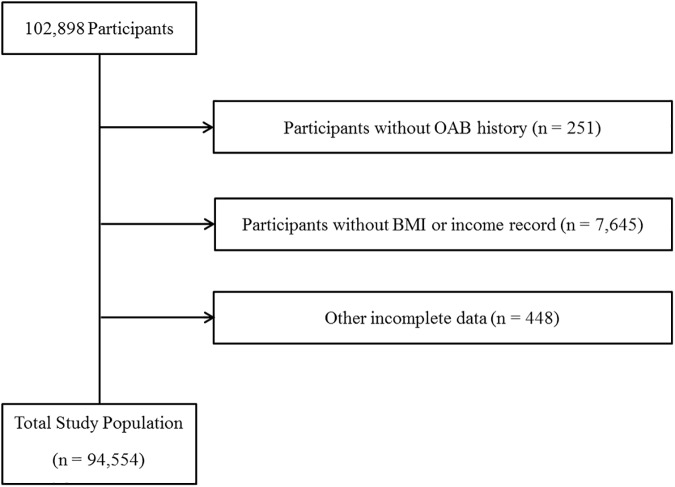
A schematic illustration of participant selection in the present study. Among a total of 102,898 participants, participants without OAB history (n = 251), BMI or income record (7,645), and other incomplete data (448) were excluded. The data for the 94,554 participants from whom complete data were obtained were analyzed.

Of a total of 102,898 male participants ranging from 19 to 107 years of age, the following participants were excluded from this study: participants who did not fill out the overactive bladder survey (251 participants); participants who did not indicate height, weight, or income record (7,645 participants); Sleep less than 3 hours (51 participants); and participants who had incomplete data related to marital status, education level, occupation, smoking, alcohol consumption history, sleep hours, stress level, hypertension, diabetes mellitus, hyperlipidemia, and cerebral stroke (397 participants). In total, 94,554 participants were included in this study (Fig 1).

### Survey

A question related to marital status, including common-law marriage, was included in the survey. To measure physical activity, participants were asked for the number of days spent during the most recent week walking more than 10 minutes. Educational level was divided into 3 groups as follows: uneducated participants and participants who had graduated from only elementary or middle schools were assigned to the “low” education group; graduates of high school comprised the “middle” group; and junior college graduates, college graduates, and participants in graduate school formed the “high” education group. Occupation was classified into 5 groups according to the level of physical activity as follows: manager, expert, specialist, clerk; service worker, salesperson; technician, mechanic, production worker, engineer; farmer, fisher, laborer, soldier; and unemployed, student. Participants under 110 cm or 30 kg were excluded from this study. Using criteria for the Asia-Pacific region [25], three body mass index (BMI, kg/m^2^) groups were devised as follows: low BMI, <18.5; normal BMI, 18.5–25; and high BMI, ≥25. Using the methods recommended by the Organization for Economic Cooperation and Development [26] (i.e., dividing household income by the square root of the number of household members), household monthly income was divided by the square root of the number of household members and categorized into lowest (0–840), low-middle (848–1,717), upper-middle (1,732–2,683), and highest (2,687–24,000) quartiles. Smoking status was divided into 3 groups: non-smoker, past smoker, and current smoker. Past smokers who had quit smoking for less than 1 year were included in the current smoker group. Alcohol consumption was divided into the following three categories: none; ≤1 time a month; 2–4 times a month; and ≥5 times a month. Amount of sleep was divided into three groups as follows: ≤6 h per day, 7–8 h per day, and ≥9 h per day. Sleep hours were surveyed as one hour interval. Participants who slept less than 3 hours per day were excluded from this study. Patients were asked whether they usually felt no stress, some stress, moderate stress, or severe stress. The participants were asked about their histories of other comorbidities, such as hypertension, diabetes mellitus, hyperlipidemia, and cerebral stroke, and participants who reported a history of any of these diseases diagnosed by a medical doctor were recorded as positive.

The OABSS, which was developed and validated in the Japanese population, was used in this study [21] (S1 Table). For the OABSS, a score ≥2 for the question “How often do you leak urine because you cannot defer the sudden desire to urinate?” and a total score ≥3 were defined as having an overactive bladder. Overactive bladder was divided into 3 groups according to the following scores: mild, total score ≤5; moderate, a total score of 6–11; severe, a total score ≥12.

### Statistical analysis

Differences in mean age and number of walking days between normal participants (control) and overactive bladder participants were compared using a linear regression analysis with complex sampling. The proportion differences of marriage, education level, occupation, income level, BMI group, smoking, alcohol consumption history, sleep hours, stress level, hypertension, diabetes mellitus, hyperlipidemia, and cerebral stroke were compared using a chi-square test with Rao-Scott correction.

To identify associations between the related factors and overactive bladder, simple and multiple logistic regression analyses with complex sampling were used. The complex sampling weighting strategy is described in detail in S1 File. In multiple logistic regression, age, walking days, marriage, education level, occupation, income level, BMI group, smoking, alcohol consumption history, sleep hours, stress level, hypertension, diabetes mellitus, hyperlipidemia, and cerebral stroke were adjusted as cofounders. Two-tailed analyses were conducted, and *P*-values less than 0.05 were considered significant. The adjusted odds ratio (AOR) and 95% confidence interval (CI) for overactive bladder were calculated. All results are presented as weighted values. The results were analyzed statistically using SPSS ver. 21.0 (IBM, Armonk, NY, USA).

## Results

**Fig 2:**
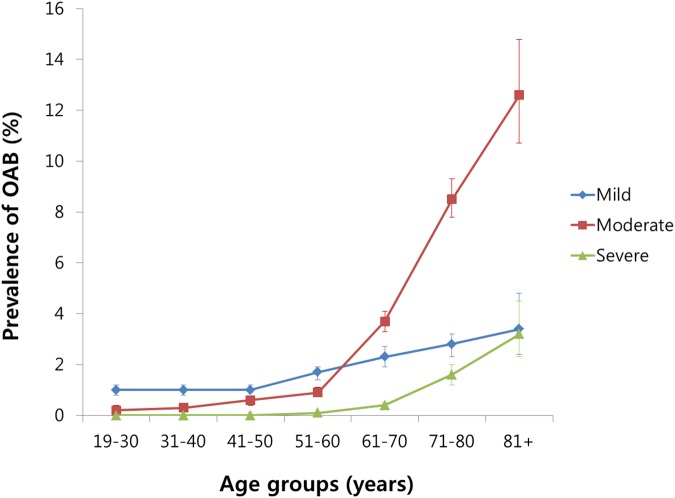
The prevalence of OAB according to age group.

The overall prevalence of OAB was 2.9% (95% CI = 2.8–3.0) and increased with age. The prevalence of OAB was the lowest in participants 19–30 years of age (1.2%) (95% CI = 1.0–1.5) and highest in individuals 81+ years of age (19.3%, 95% CI = 17.0–21.8). From 19–30 years of age through 51–60 years of age, the prevalence of mild, moderate, and severe OAB was relatively stationary, and mild OAB was more frequent than moderate or severe OAB. However, all degrees of OAB steeply increased after 61 years of age, especially moderate OAB. Consequently, moderate OAB was more prevalent than mild OAB in the 61+ years age groups (Fig 2, S2 Table).

As the prevalence of OAB increased with age, the mean age of OAB (58.6 years of age, 95% CI = 57.8–59.4) was higher than the normal control group (44.2 years of age, 95% CI = 44.1–44.3) (P<0.001) (Table 1). The percentage of married subjects who experienced OAB was 3.4% (95% CI = 3.2–3.5), which was significantly more than the 1.5% (95% CI = 1.3–1.7) of unmarried subjects who experienced OAB (P<0.001). The prevalence of OAB increased as education level decreased (P<0.001). Types of occupations showed significant differences between the control and OAB groups (P<0.001). Lower income levels showed significant associations with OAB (P<0.001). Other retrieved variables, including BMI; smoking; alcohol; sleep time; level of stress; and medical history of hypertension, diabetes mellitus, hyperlipidemia, and/or cerebral stroke, were significantly different between the control and OAB groups (all, P<0.001).

**Table 1:** General characteristics of participants.

		Control	Overactive bladder	P-value
Number				
	N	90,944	3,610	
	%	97.1 (97.0–97.2)	2.9 (2.8–3.0)	
Age (year)		44.2 (44.1–44.3)	58.6 (57.8–59.4)	<0.001*
Walking day (d)		4.2	4.2	0.625
Marriage (%, 95% CI)				<0.001†
	Yes	73.9 (73.5–74.3)	86.8 (84.9–88.5)	
	No	26.1 (25.7–26.5)	13.2 (11.5–15.1)	
Education (%, 95% CI)				<0.001†
	Low	15.2 (15.0–15.5)	38.8 (36.7–40.9)	
	Middle	31.7 (31.3–32.1)	27.8 (25.8–29.9)	
	High	53.1 (52.7–53.5)	33.4 (31.1–35.7)	
Occupation (%, 95% CI)				<0.001†
	Manager, Expert, Specialist, Clerk	32.9 (32.5–33.3)	14.7 (13.0–16.5)	
	Service worker, salesperson	5.8 (5.6–6.0)	3.1 (2.4–4.0)	
	Technician, Mechanics, Production worker, Engineer	23.9 (23.5–24.2)	17.1 (15.3–19.0)	
	Farmer, Fisher, Laborer, Soldier	14.6 (14.3–14.8)	18.6 (17.1–20.1)	
	Student	8.5 (8.3–8.7)	4.7 (3.8–5.9)	
	Unemployed	14.4 (14.1–14.6)	41.9 (39.7–44.1)	
Income (%, 95% CI)				<0.001†
	Lowest	10.0 (9.7–10.2)	27.6 (25.8–29.5)	
	Low-middle	24.7 (24.3–25.1)	30.0 (27.9–32.0)	
	Upper-middle	31.2 (30.7–31.6)	20.8 (18.9–22.7)	
	Highest			
BMI (kg/m^2^) (%, 95% CI)				<0.001†
	<18.5	2.3 (2.1–2.4)	5.3 (4.5–6.3)	
	≥18.5, <25	67.2 (66.8–67.6)	68.4 (66.3–70.5)	
	≥25	30.6 (30.2–30.9)	26.2 (24.2–28.4)	
Smoking (%, 95% CI)				<0.001†
	None	26.3 (25.9–26.7)	19.7 (17.9–21.7)	
	Past smoker	28.4 (28.1–28.8)	43.1 (40.0–45.3)	
	Current smoker	45.3 (44.8–45.7)	37.2 (35.0–39.4)	
Alcohol (%, 95% CI)				<0.001†
	None	15.2 (14.9–15.5)	29.3 (27.4–31.2)	
	≤ 1 time a month	18.5 (18.2–18.9)	16.0 (14.4–17.8)	
	2–4 times a month	29.8 (29.4–30.2)	20.3 (18.5–22.2)	
	≥ 5 times a month	36.5 (36.1–36.9)	34.5 (32.4–36.6)	
Sleep (hours) (%, 95% CI)				<0.001†
	≤ 6h	45.7 (45.2–46.1)	47.3 (45.1–49.6)	
	7-8h	51.1 (50.7–51.5)	44.7 (42.5–46.9)	
	≥ 9h	3.3 (3.1–3.4)	8.0 (6.9–9.2)	
Stress (%, 95% CI)				<0.001†
	No	16.7 (16.4–17.0)	19.0 (17.5–20.7)	
	Some	55.4 (55.0–55.8)	44.7 (42.5–47.0)	
	Moderate	24.2 (23.9–24.6)	28.6 (26.6–30.7)	
	Severe	3.7 (3.5–3.8)	7.6 (6.4–9.0)	
Hypertension (%, 95% CI)				<0.001†
	Yes	16.6 (16.3–16.9)	35.9 (33.8–38.1)	
	No	83.4 (83.1–83.7)	64.1 (61.9–66.2)	
Diabetes mellitus (%, 95% CI)				<0.001†
	Yes	6.5 (6.3–6.7)	17.6 (16.1–19.3)	
	No	93.5 (93.3–93.7)	82.4 (80.7–83.9)	
Hyperlipidemia (%, 95% CI)				<0.001†
	Yes	9.9 (9.7–10.2)	17.1 (15.4–18.9)	
	No	90.1 (89.8–90.3)	82.9 (81.1–84.6)	
Cerebral Stroke (%, 95% CI)				<0.001†
	Yes	1.1 (1.0–1.2)	8.2 (7.2–9.4)	
	No	98.9 (98.8–99.0)	91.8 (90.6–92.8)	

* Linear regression analysis with complex sampling, Significance at P < 0.05

† Chi-square test with Rao-Scott correction, Significance at P < 0.05

CI: confidence interval

All of the variables showed statistical significance by univariate analysis with calculated odds ratios in unadjusted and full adjusted models. When considering age, the odds ratios of OAB for an increase in 10-year intervals were 1.80 (95% CI = 1.73–1.87, P<0.001) and 1.70 (95% CI = 1.61–1.80, P<0.001) by simple and multiple regression analyses, respectively (Table 2). Although married subjects showed a higher odds ratio of OAB by simple regression analysis (OR = 2.32, 95% CI = 1.98–2.72, P<0.001), they showed a lower odds ratio of OAB than the control group when adjusting for other variables (AOR = 0.59, 95% CI = 0.48–0.72, P<0.001). Higher levels of education were related to OAB in a dose-dependent manner in unadjusted models (P<0.001). However, only the middle level of education showed fewer reports of OAB than the high education group in adjusted models (AOR = 0.83, 95% CI = 0.72–0.96, P<0.001). The prevalence of OAB was significantly different according to the types of occupations in both unadjusted and adjusted models (all of P<0.001). Compared to the manager, expert, specialist, and clerk group, other occupation groups showed significantly higher odds ratios (P<0.001) in the following order: unemployed (AOR = 1.90, 95% CI = 1.55–2.32); student (AOR = 1.17, 95% CI = 0.89–1.55); farmer, fisher, laborer, and soldier (AOR = 1.46, 95% CI = 1.20–1.79); technician, mechanic, production worker, and engineer (AOR = 1.45, 95% CI = 1.19–1.76); and service worker and salesperson (AOR = 1.22, 95% CI = 0.91–1.65). Compared to the highest income level, the lower income levels were related to increased OAB in the unadjusted model (P<0.001). After adjusting for other factors, only the lowest income level showed a significant association with OAB (AOR = 1.25, 95% CI = 1.06–1.49, P = 0.002). Being underweight was significantly associated with OAB (AOR = 1.29, 95% CI = 1.08–1.55, P = 0.018), while the obese group was not significantly related to OAB (AOR = 0.99, 95% CI = 0.88–1.11, P = 0.018). Smoking and alcohol were significantly related to OAB in the simple regression analysis, but statistical significances were not maintained in the multiple regression analysis (P = 0.120 and 0.418 for smoking and alcohol, respectively). Both lack of sleep and excessive sleep were significantly associated with OAB (AOR = 1.13, 95% CI = 1.02–1.25 for ≤6 hours of sleep time and AOR = 1.53, 95% CI = 1.27–1.86 for ≥9 hours of sleep time, P<0.001). Level of stress was significantly correlated with OAB in a dose-dependent manner (AOR [95% CI] = 3.91 [3.13–4.89] > 2.16 [1.88–2.48] > 1.39 [1.23–1.57] for severe stress > moderate stress > some stress, respectively, P<0.001). A medical history of diabetes mellitus (AOR = 1.26, 95% CI = 1.11–1.43, P<0.001), hyperlipidemia (AOR = 1.30, 95% CI = 1.13–1.49, P<0.001), and cerebral stroke (AOR = 2.17, 95% CI = 1.81–2.61, P<0.001) showed significant associations with OAB.

**Table 2:** Odd ratios of possible risk factors for overactive bladder using simple and multiple logistic regression analyses with complex sampling.

		Simple Regression			Multiple Regression		
		OR	95% CI	P-value	AOR	95% CI	P-value
Age (10 years)		1.80	1.73–1.87	<0.001*	1.70	1.61–1.80	<0.001*
Walking Day		1.00	0.98–1.01	0.624	1.00	0.99–1.02	0.584
Marriage				<0.001*			<0.001*
	Yes	2.32	1.98–2.72		0.59	0.48–0.72	
	No	1			1		
Education				<0.001*			0.004*
	Low	4.05	3.63–4.53		1.01	0.86–1.16	
	Middle	1.40	1.23–1.58		0.83	0.72–0.96	
	High	1			1		
Occupation				<0.001*			<0.001*
	Manager, Expert, Specialist, Clerk	1			1		
	Service worker, salesperson	1.19	0.89–1.60		1.22	0.91–1.65	
	Technician, Mechanics, Production worker, Engineer	1.61	1.35–1.92		1.45	1.19–1.76	
	Farmer, Fisher, Laborer, Soldier	2.86	2.44–3.35		1.46	1.20–1.79	
	Student	1.24	0.95–1.63		1.17	0.89–1.55	
	Unemployed	6.56	5.65–7.61		1.90	1.55–2.32	
Income				<0.001*			0.002*
	Lowest	4.37	3.82–5.00		1.25	1.06–1.49	
	Low-middle	1.91	1.67–2.19		1.06	0.91–1.24	
	Upper-middle	1.05	0.90–1.22		0.94	0.80–1.10	
	Highest	1			1		
BMI (kg/m^2^)				<0.001*			0.018*
	<18.5	2.31	1.94–2.76		1.29	1.08–1.55	
	≥18.5, <25	1			1		
	≥25	0.84	0.76–0.94		0.99	0.88–1.11	
Smoking				<0.001*			0.120
	None	1			1		
	Past smoker	2.02	1.78–2.29		1.15	1.01–1.32	
	Current smoker	1.09	0.96–1.25		1.10	0.96–1.27	
Alcohol				<0.001*			0.418
	None	1			1		
	≤ 1 time a month	0.45	0.39–0.52		0.91	0.78–1.06	
	2–4 times a month	0.35	0.31–0.40		0.90	0.78–1.04	
	≥ 5 times a month	0.49	0.44–0.55		0.95	0.84–1.07	
Sleep (hours)				<0.001*			<0.001*
	≤ 6h	1.18	1.08–1.30		1.13	1.02–1.25	
	7-8h	1			1		
	≥ 9h	2.80	2.36–3.33		1.53	1.27–1.86	
Stress				<0.001*			<0.001*
	No	1			1		
	Some	0.71	0.63–0.79		1.39	1.23–1.57	
	Moderate	1.04	0.91–1.18		2.16	1.88–2.48	
	Severe	1.81	1.48–2.23		3.91	3.13–4.89	
Hypertension				<0.001*			0.061
	Yes	2.81	2.56–3.09		1.11	1.00–1.24	
	No	1			1		
Diabetes mellitus				<0.001*			<0.001*
	Yes	3.08	2.74–3.45		1.26	1.11–1.43	
	No	1			1		
Hyperlipidemia				<0.001*			<0.001*
	Yes	1.87	1.65–2.11		1.30	1.13–1.49	
	No	1			1		
Cerebral Stroke				<0.001*			<0.001*
	Yes	8.03	6.84–9.43		2.17	1.81–2.61	
	No	1			1		

* Significance at P < 0.05.

Adjusted variables in multiple logistic regression: Age, walking days, marriage, education, occupation, income, BMI, smoking, alcohol, sleep, stress, hypertension, diabetes mellitus, hyperlipidemia, and cerebral stroke.

## Discussion

The prevalence of OAB in Korean men was 2.9%, which was somewhat lower than the 10 to 20% reported in previous studies [2–4, 16]. These variations in the prevalence of OAB might be explained by variations in survey questionnaires for OAB, methods of conducting a survey, and characteristics of the study populations. Although some variations were observed in the overall prevalence of OAB across age groups, the increasing tendency of OAB with aging was comparable among the studies [2, 16]. In accordance with previous reports, the present study showed that the prevalence of OAB was markedly increased after 60 years of age, especially moderate OAB. The prevalence of OAB in individuals ≥81 years of age was 19.3% in the present study, which was comparable to the findings in previous studies [2–4, 16]. In addition to age, several variables showed significant associations with OAB in this study. Unmarried status; types of occupation; being underweight; inadequate sleep time; stress; and a medical history of diabetes mellitus, hyperlipidemia, and/or cerebral stroke were significantly related with OAB when adjusted for other associated factors of OAB.

Advanced age was associated with OAB. Various studies, including this study, have shown that the prevalence of OAB precipitously increases after middle age [3, 5]. Several aging-associated processes of the bladder, including reduced adaptive changes of functional capacity to maintain consistency of voiding frequency, involuntary detrusor overactivity, and structural changes such as disorientation of muscles, were suggested to contribute to urinary incontinence in the elderly [27]. Accumulated oxidative stress and pro-inflammatory status may also influence the decline in bladder function [28, 29]. Moreover, with aging, an increase in small vessel disease in the frontal cortex, which functions as a micturition center, can cause OAB [30].

Being underweight showed a significant association with OAB in this study. Some variations in the effects of BMI on OAB exist among previous studies. Many studies have reported that high BMI is associated with OAB because of the increase in bladder pressure in obesity [5]. However, some studies reported no significant association between being overweight and OAB [13]. It is presumed that the detrimental effects of obesity may not be evident in the Korean population because Koreans rarely exhibit severe obesity to such an extent that bladder pressure is increased. On the other hand, comorbidities related to being underweight, such as renal disease and tumorous conditions, could have had more influence on OAB in this study [31, 32].

Participants with a medical history of diabetes mellitus, hyperlipidemia, and/or cerebral stroke demonstrated significant associations with OAB. Several previous studies reported associations of medical comorbidities with OAB, including metabolic syndrome and stroke [8, 12]. Diabetes was especially related to bladder dysfunction, which occurs in 80% of diabetic patients [29]. These associations were suggested to depend on the deregulation of the autonomic nervous system and subsequent bladder dysfunction in compromised diabetes patients [12, 30].

Interestingly, several variables that were reported as risk factors for OAB in previous studies demonstrated discrepancies between simple and multiple regression analyses in the present study. In contrast to previous studies [5], our data suggested that the prevalence of OAB was lower in married men than unmarried men when adjusted for other possible associated factors of OAB, although unadjusted models demonstrated the opposite result and showed a higher prevalence of OAB in married men. This positive association between OAB and marriage in the simple logistic regression analysis was presumed to be related to various confounders, including age. Because the average ages of married men were older than unmarried men, age might be a confounder in the unadjusted model. In addition, hidden links or shared risk factors could explain this association between OAB and unmarried status. LUTS are known to be associated with diminished sexual function, including erectile dysfunction [33, 34]. Sexual dysfunction is generally more common in unmarried subjects [35]. Thus, OAB may be associated with unmarried status because both OAB and being unmarried are associated with sexual dysfunction.

Smoking and alcohol did not show significant associations with OAB in our multiple regression models. On the contrary, several previous studies demonstrated a higher incidence of OAB in subjects who drank alcohol or smoked [36–38]. The positive association between smoking and OAB is presumed to be related to poor compliance to the medication of smokers [39]. Moreover, nicotine may promote a release of neurotransmitters from the central and peripheral nervous systems, causing detrusor overactivity [38]. However, other studies did not show significant associations between smoking and OAB [40, 41]. Gender differences related to smoking and OAB were suggested; women who smoked demonstrated a significant association with OAB, while men did not show a significant association between smoking and OAB [42]. In women, nicotine has an anti-estrogenic effect, which affects the bladder wall strength and detrusor instability [38, 42]. Because our survey was based on the male population, hormonal and neurogenic effects of smoking might not have been as evident in this study. Furthermore, the detrimental effects of alcohol and smoking might differ according to the amounts and frequency of intake. We did not consider the level of intake, which might have resulted in statistical insignificances between alcohol or smoking and OAB in this study. Moreover, we considered various socioeconomic and medical conditions associated with lifestyles related to smoking and alcohol; thus, the confounding effects associated with smoking and alcohol could be minimized in the multiple logistic regression analyses.

Education and economic levels are known to be related factors of OAB [2, 5]. However, these variables showed attenuated associations with OAB in the adjusted models compared to unadjusted models in this study. Specifically, although education levels showed significant associations with OAB in a dose-dependent manner in the unadjusted model, the middle level of the education group was the only significantly different group in OAB compared to the lowest education level group after adjusting for other factors. Similarly, only the lowest income group showed a significantly higher prevalence of OAB than the highest income group in the adjusted model. Because the levels of education and income were subdivided, the differences in OAB according to education and economic levels might be visualized in the present study but not in previous studies in which these variables were dichotomized [2, 5]. Moreover, the alleviated associations of income and education levels with OAB might reflect effects of other associated factors, such as occupation. Occupation showed consistently significant associations with OAB in both unadjusted and adjusted models. Thus, differences in OAB according to education and income levels in unadjusted models might have originated from the differences in occupations depending on socio-economic status. The income and education levels might have shown significant differences in OAB in previous studies because those studies did not consider possible associated factors of OAB, such as types of occupations [2, 5].

Occupation showed significantly different associations with OAB. A recent study suggested that the occupation of manual workers was associated with OAB owing to the physical activities of this occupation, which increases abdominal pressure, thereby weakening the pelvic floor [5]. Another recent study showed that government employees were negatively related to OAB, although the statistical significance was not maintained in the multivariable analysis [43]. The authors presumed that an unstable occupational status might have a detrimental emotional effect, which may result in OAB. Our sub-classifications of occupations encompassed various types of occupations, thereby reflecting both physical and psychological factors related to OAB. Healthy behavior and better lifestyles of higher socio-economic subjects contributed in part to their lower prevalence of OAB [5, 43].

Inadequate sleep was correlated with OAB in the present study in accordance with previous studies [5]. OAB could hinder sleep hygiene and cause a lack of sleep [5]. Excessive sleep time as well as the lack of sleep could imply the poor quality of sleep. A recent prospective study demonstrated that the long time-in-bed was associated with poor perceived sleep quality and more negative mood [44]. Moreover, emotional problems that often accompany sleep problems might affect bladder function. Indeed, bladder function is somewhat controlled by emotion. It is known that depression and anxiety cause bladder dysfunction [15]. Moreover, this study and prior studies demonstrated that stressful events had a negative impact on voiding function [45]. Furthermore, OAB patients are more susceptible to anxiety and depression symptoms [46–48]. Limitations of a cross-sectional study design include a lack of delineation of the causal relationships between OAB and stress factors, and further study with a prospective study design is needed to reveal these causal associations.

Other than a cross-sectional study design, several points should be considered in the present study. The self-reported survey of OAB limited the interpretation of our results. Recall bias was inevitable. Although we comprehensively included numerous variables to minimize the possible confounding effects, it was still possible that hidden confounders were missed in our analyses. However, the present study conducted multivariable analyses of various factors. Moreover, a large number of participants was selected based on the validated random selection strategies; therefore, a representative nation-wide population could be collected. All of these factors strengthened the statistical power and reliability of our data. A standardized validated survey with OABSS was another advantage of this study.

## Conclusion

Approximately 2.9% of adult Korean men complained of OAB based on the OABSS. The prevalence of OAB increased with age. Moreover, various physical factors, including being underweight; a medical history of diabetes mellitus, hyperlipidemia, and/or cerebral stroke; socio-economic factors of unmarried status, occupation, education and income level; and psychologic factors of inadequate sleep time and stress, were significantly related to OAB.

## Supporting information

S1 File The analytic methods of weighting. (DOCX)

S1 Table Overactive bladder symptom score. (DOCX)

S2 Table Prevalence of OAB according to severity. (DOCX)

## References

[1] Barozzi L, Valentino M, Menchi I, Pavlica P. Clinical uroradiology: the standardisation of terminology for lower urinary tract function and dysfunction. Radiol Med. 2010;115(2):272–286. doi: 10.1007/s11547-010-0509-0 20101525 10.1007/s11547-010-0509-0

[2] Stewart WF, Van Rooyen JB, Cundiff GW, Abrams P, Herzog AR, Corey R, et al Prevalence and burden of overactive bladder in the United States. World J Urol. 2003;20(6):327–336. doi: 10.1007/s00345-002-0301-4 12811491 10.1007/s00345-002-0301-4

[3] Irwin DE, Milsom I, Kopp Z, Abrams P, Cardozo L. Impact of overactive bladder symptoms on employment, social interactions and emotional well-being in six European countries. BJU Int. 2006;97(1):96–100. doi: 10.1111/j.1464-410X.2005.05889.x 16336336 10.1111/j.1464-410X.2005.05889.x

[4] Milsom I, Abrams P, Cardozo L, Roberts RG, Thuroff J, Wein AJ. How widespread are the symptoms of an overactive bladder and how are they managed? A population-based prevalence study. BJU Int. 2001;87(9):760–766 11412210 10.1046/j.1464-410x.2001.02228.x

[5] Wang Y, Xu K, Hu H, Zhang X, Wang X, Na Y, et al Prevalence, risk factors, and impact on health related quality of life of overactive bladder in China. Neurourol Urodyn. 2011;30(8):1448–1455. doi: 10.1002/nau.21072 21826714 10.1002/nau.21072

[6] Coyne KS, Margolis MK, Kopp ZS, Kaplan SA. Racial differences in the prevalence of overactive bladder in the United States from the epidemiology of LUTS (EpiLUTS) study. Urology. 2012;79(1):95–101. doi: 10.1016/j.urology.2011.09.010 22055692 10.1016/j.urology.2011.09.010

[7] Lee KS, Choo MS, Seo JT, Oh SJ, Kim HG, Ng K, et al Impact of overactive bladder on quality of life and resource use: results from Korean Burden of Incontinence Study (KOBIS). Health Qual Life Outcomes. 2015;13:89 doi: 10.1186/s12955-015-0274-9 26113125 10.1186/s12955-015-0274-9 PMC4480453

[8] Sacco E, Bientinesi R, Marangi F, D'Addessi A, Racioppi M, Gulino G, et al Overactive bladder syndrome: the social and economic perspective]. Urologia. 2011;78(4):241–256. doi: 10.5301/RU.2011.8886 22237808 10.5301/RU.2011.8886

[9] Kinsey D, Pretorius S, Glover L, Alexander T. The psychological impact of overactive bladder: A systematic review. J Health Psychol. 2016;21(1):69–81. doi: 10.1177/1359105314522084 24591118 10.1177/1359105314522084

[10] Coyne KS, Sexton CC, Clemens JQ, Thompson CL, Chen CI, Bavendam T, et al The impact of OAB on physical activity in the United States: results from OAB-POLL. Urology. 2013;82(4):799–806. doi: 10.1016/j.urology.2013.05.035 23953610 10.1016/j.urology.2013.05.035

[11] Wu EQ, Birnbaum H, Marynchenko M, Mareva M, Williamson T, Mallett D. Employees with overactive bladder: work loss burden. J Occup Environ Med. 2005;47(5):439–446. 15891521 10.1097/01.jom.0000161744.21780.c1

[12] Coyne KS, Sexton CC, Thompson CL, Clemens JQ, Chen CI, Bavendam T, et al Impact of overactive bladder on work productivity. Urology. 2012;80(1):97–103. doi: 10.1016/j.urology.2012.03.039 22748868 10.1016/j.urology.2012.03.039

[13] Bunn F, Kirby M, Pinkney E, Cardozo L, Chapple C, Chester K, et al Is there a link between overactive bladder and the metabolic syndrome in women? A systematic review of observational studies. Int J Clin Pract. 2015;69(2):199–217. doi: 10.1111/ijcp.12518 25495905 10.1111/ijcp.12518

[14] Kumar V, Cross RL, Chess-Williams R, Chapple CR. Recent advances in basic science for overactive bladder. Curr Opin Urol. 2005;15(4):222–226 15928509 10.1097/01.mou.0000172393.52857.92

[15] Sakakibara R, Ito T, Yamamoto T, Uchiyama T, Yamanishi T, Kishi M, et al Depression, anxiety and the bladder. Low Urin Tract Symptoms. 2013;5(3):109–120. doi: 10.1111/luts.12018 26663445 10.1111/luts.12018

[16] Lee YS, Lee KS, Jung JH, Han DH, Oh SJ, Seo JT, et al Prevalence of overactive bladder, urinary incontinence, and lower urinary tract symptoms: results of Korean EPIC study. World J Urol. 2011;29(2):185–190. doi: 10.1007/s00345-009-0490-1 19898824 10.1007/s00345-009-0490-1

[17] Kirby MG, Wagg A, Cardozo L, Chapple C, Castro-Diaz D, de Ridder D, et al Overactive bladder: is there a link to the metabolic syndrome in men? Neurourol Urodyn. 2010;29(8):1360–1364. doi: 10.1002/nau.20892 20589717 10.1002/nau.20892

[18] Dallosso HM, McGrother CW, Matthews RJ, Donaldson MM, Leicestershire MRC, Incontinence Study Group. The association of diet and other lifestyle factors with overactive bladder and stress incontinence: a longitudinal study in women. BJU Int. 2003;92(1):69–77. 12823386 10.1046/j.1464-410x.2003.04271.x

[19] Choo MS, Ku JH, Park CH, Lee YS, Lee KS, Lee JG, et al Prevalence of nocturia in a Korean population aged 40 to 89 years. Neurourol Urodyn. 2008;27(1):60–64. doi: 10.1002/nau.20458 17565726 10.1002/nau.20458

[20] Choo MS, Ku JH, Oh SJ, Lee KS, Paick JS, Seo JT, et al Prevalence of urinary incontinence in Korean women:an epidemiologic survey. Int Urogynecol J Pelvic Floor Dysfunct. 2007;18(11):1309–1315. doi: 10.1007/s00192-007-0322-z 17912572 10.1007/s00192-007-0322-z

[21] Homma Y, Yoshida M, Seki N, Yokoyama O, Kakizaki H, Gotoh M, et al Symptom assessment tool for overactive bladder syndrome—overactive bladder symptom score. Urology. 2006;68(2):318–323. doi: 10.1016/j.urology.2006.02.042 16904444 10.1016/j.urology.2006.02.042

[22] Korea Centers for Disease Control and Prevention, The Community Health Survey. 2013 [cited 2013 Oct 5]. Available from: https://chs.cdc.go.kr.

[23] Rim H, Kim H, Lee K, Chang S, Hovell MF, Kim YT, et al Validity of self-reported healthcare utilization data in the Community Health Survey in Korea. J Korean Med Sci. 2011;26(11):1409–1414. doi: 10.3346/jkms.2011.26.11.1409 22065895 10.3346/jkms.2011.26.11.1409 PMC3207042

[24] Oh DH, Kim SA, Lee HY, Seo JY, Choi BY, Nam JH. Prevalence and correlates of depressive symptoms in Korean adults: results of a 2009 Korean community health survey. J Korean Med Sci. 2013;28(1):128–135. doi: 10.3346/jkms.2013.28.1.128 23341723 10.3346/jkms.2013.28.1.128 PMC3546091

[25] International Obesity TaskForce. The Asia-Pacific perspective: Redefining obesity and its treatment. 2000 2000 [cited 2014 20 Nov]. Available from: http://www.wpro.who.int/nutrition/documents/docs/Redefiningobesity.pdf.

[26] OECD Project on Income Distribution and Poverty. What are equivalence scales? 2009 [cited 2014 20 Nov]. Available from: http://www.oecd.org/eco/growth/OECD-Note-EquivalenceScales.pdf.

[27] Camoes J, Coelho A, Castro-Diaz D, Cruz F. Lower urinary tract symptoms and aging: the impact of chronic bladder ischemia on overactive bladder syndrome. Urol Int. 2015;95(4):373–379. doi: 10.1159/000437336 26466093 10.1159/000437336

[28] Kregel KC, Zhang HJ. An integrated view of oxidative stress in aging: basic mechanisms, functional effects, and pathological considerations. Am J Physiol Regul Integr Comp Physiol. 2007;292(1):R18–36. doi: 10.1152/ajpregu.00327.2006 16917020 10.1152/ajpregu.00327.2006

[29] Chung HY, Sung B, Jung KJ, Zou Y, Yu BP. The molecular inflammatory process in aging. Antioxid Redox Signal. 2006;8(3–4):572–581. doi: 10.1089/ars.2006.8.572 16677101 10.1089/ars.2006.8.572

[30] Sakakibara R, Panicker J, Fowler CJ, Tateno F, Kishi M, Tsuyusaki Y, et al Is overactive bladder a brain disease? The pathophysiological role of cerebral white matter in the elderly. Int J Urol. 2014;21(1):33–38. doi: 10.1111/iju.12288 24118122 10.1111/iju.12288

[31] Migita K, Takayama T, Matsumoto S, Wakatsuki K, Tanaka T, Ito M, et al Impact of being underweight on the long-term outcomes of patients with gastric cancer. Gastric Cancer. 2015;19(3):735–743. doi: 10.1007/s10120-015-0531-y 26298184 10.1007/s10120-015-0531-y

[32] Echeverria-Esnal D, Retamero A, Pardos SL, Grau S. Severe thrombocytopenia caused by linezolid poisoning in an underweight critically ill patient with renal impairment treated with the recommended doses. Enferm Infecc Microbiol Clin. 2016;34(3):213–214. doi: 10.1016/j.eimc.2015.06.012 26211841 10.1016/j.eimc.2015.06.012

[33] Tannenbaum C. Associations between urinary symptoms and sexual health in older adults. Clin Geriatr Med. 2015;31(4):581–590. doi: 10.1016/j.cger.2015.06.007 26476117 10.1016/j.cger.2015.06.007

[34] Korneyev IA, Alexeeva TA, Al-Shukri SH, Bernikov AN, Erkovich AA, Kamalov AA, et al Prevalence and risk factors for erectile dysfunction and lower urinary tract symptoms in Russian Federation men: analysis from a national population-based multicenter study. Int J Impot Res. 2016;28(2):74–79. doi: 10.1038/ijir.2016.8 26865104 10.1038/ijir.2016.8

[35] McCabe MP, Sharlip ID, Lewis R, Atalla E, Balon R, Fisher AD, et al Risk factors for sexual dysfunction among women and men: a consensus statement from the Fourth International Consultation on Sexual Medicine 2015. J Sex Med. 2016;13(2):153–167. doi: 10.1016/j.jsxm.2015.12.015 26953830 10.1016/j.jsxm.2015.12.015

[36] Hirayama A, Torimoto K, Mastusita C, Okamoto N, Morikawa M, Tanaka N, et al Risk factors for new-onset overactive bladder in older subjects: results of the Fujiwara-kyo study. Urology. 2012;80(1):71–76. doi: 10.1016/j.urology.2012.04.019 22626577 10.1016/j.urology.2012.04.019

[37] de Boer TA, Slieker-ten Hove MC, Burger CW, Vierhout ME. The prevalence and risk factors of overactive bladder symptoms and its relation to pelvic organ prolapse symptoms in a general female population. Int Urogynecol J. 2011;22(5):569–575. doi: 10.1007/s00192-010-1323-x 21104400 10.1007/s00192-010-1323-x PMC3072516

[38] Madhu C, Enki D, Drake MJ, Hashim H. The functional effects of cigarette smoking in women on the lower urinary tract. Urol Int. 2015;95(4):478–482. doi: 10.1159/000438928 26452108 10.1159/000438928

[39] Brubaker L, Fanning K, Goldberg EL, Benner JS, Trocio JN, Bavendam T, et al Predictors of discontinuing overactive bladder medications. BJU Int. 2010;105(9):1283–1290. doi: 10.1111/j.1464-410X.2009.09035.x 19912189 10.1111/j.1464-410X.2009.09035.x

[40] Cheung WW, Blank W, Borawski D, Tran W, Bluth MH. Prevalence of overactive bladder, its under-diagnosis, and risk factors in a male urologic veterans population. Int J Med Sci. 2010;7(6):391–394. 21103074 10.7150/ijms.7.391 PMC2990074

[41] Jo JK, Lee S, Kim YT, Choi HY, Kim SA, Choi BY, et al Analysis of the risk factors for overactive bladder on the basis of a survey in the community. Korean J Urol. 2012;53(8):541–546. doi: 10.4111/kju.2012.53.8.541 22949998 10.4111/kju.2012.53.8.541 PMC3427838

[42] Maserejian NN, Kupelian V, Miyasato G, McVary KT, McKinlay JB. Are physical activity, smoking and alcohol consumption associated with lower urinary tract symptoms in men or women? Results from a population based observational study. J Urol. 2012;188(2):490–495. doi: 10.1016/j.juro.2012.03.128 22704109 10.1016/j.juro.2012.03.128 PMC3427389

[43] Kim JH, Ham BK, Shim SR, Lee WJ, Kim HJ, Kwon SS, et al The association between the self-perception period of overactive bladder symptoms and overactive bladder symptom scores in a non-treated population and related sociodemographic and lifestyle factors. Int J Clin Pract. 2013;67(8):795–800. doi: 10.1111/ijcp.12080 23869680 10.1111/ijcp.12080

[44] Bei B, Manber R, Allen NB, Trinder J, Wiley JF. Too long, too short, or too variable? Sleep intraindividual variability and its associations with perceived sleep quality and mood in adolescents during naturalistically unconstrained sleep. Sleep. 2016. 10.1093/sleep/zsw067 28364491

[45] Chang SJ, Chiang IN, Lin CD, Hsieh CH, Yang SS. Obese children at higher risk for having overactive bladder symptoms: a community-based study. Neurourol Urodyn. 2015;34(2):123–127. doi: 10.1002/nau.22532 24273112 10.1002/nau.22532

[46] Drossaerts J, Vrijens D, Leue C, Schilders I, Van Kerrebroeck P, van Koeveringe G. Screening for depression and anxiety in patients with storage or voiding dysfunction: a retrospective cohort study predicting outcome of sacral neuromodulation. Neurourol Urodyn. 2015 9 9 Available from: http://www.ncbi.nlm.nih.gov/pubmed/26351817. 10.1002/nau.22871 26351817

[47] Kabra AT, Feustel PJ, Kogan BA. Screening for depression and anxiety in childhood neurogenic bladder dysfunction. J Pediatr Urol. 2015;11(2):75e1–e7. 25824880 10.1016/j.jpurol.2014.11.017

[48] Milsom I, Kaplan SA, Coyne KS, Sexton CC, Kopp ZS. Effect of bothersome overactive bladder symptoms on health-related quality of life, anxiety, depression, and treatment seeking in the United States: results from EpiLUTS. Urology. 2012;80(1):90–96. doi: 10.1016/j.urology.2012.04.004 22748867 10.1016/j.urology.2012.04.004

