# Supplementary material for: Analysis of the prevalence and associated factors of overactive bladder in adult Korean men
Source: PLoS One. 2017 Apr 13;12(4):e0175641. doi: 10.1371/journal.pone.0175641 (PMC5391112; doi:10.1371/journal.pone.0175641)
Supplement: S1 File — (DOCX) [file pone.0175641.s001.docx]

**S1 File The analytic methods of weighting**

In this study, the participants were weighted using this method to represent mother populations. Weighted values were calculated using household weight, personal weight, and adjusted weight.

(1) Household weight were calculated as below

W_hi_ = M_hi_/n_hi_ = e_hi_ * N_hi_ /n_hi_

N_hi_ = i type household number in h (tong/ban/ri) descrict

M_hi_ = i type appropriate household number in h (tong/ban/ri) descrict

n_hi_ = i type survey household number in in h (tong/ban/ri) descrict

e_hi_ = i type appropriate survey household number in h (tong/ban/ri) descrict

(2) Personal weight were calculated as below

W_hjk_ = W_hjk_ * P_hjk_/p_hjk_

h: (tong/ban/ri) descrict

j: smple are

k; household

l; the number of household member

P_hjk_: the number of household ≥ 19 years old

p_hjk_: the number of survey household ≥ 19 years old

(3) Adjusted weight were calculated as below to adjust population structure according to the resident registration number

W`_hijkl_ = BF_d_ * W_hijkl_

BF_d_ = d (the number of population according to the resident registration number according to age and sex)/(∑_h_∑_i_∑_jkl_ W_hijkl_ I _d_ ∈_according to age, sex_)
