# Supplementary material for: Analysis of the prevalence and associated factors of overactive bladder in adult Korean men
Source: PLoS One. 2017 Apr 13;12(4):e0175641. doi: 10.1371/journal.pone.0175641 (PMC5391112; doi:10.1371/journal.pone.0175641)
Supplement: S2 Table — (DOCX) [file pone.0175641.s003.docx]

**S2 Table** Prevalence of OAB according to severity

| OAB | Age (years old) | | | | | | |
| --- | --- | --- | --- | --- | --- | --- | --- |
|  | 19-30 | 31-40 | 41-50 | 51-60 | 61-70 | 71-80 | 81+ |
| No (n, %, 95% CI*) | 11,982 (98.8, 98.5-99.0) | 16,265 (98.8, 98.6-99.0) | 19,647 (98.4, 98.1-98.6) | 18,698 (97.4, 98.1-98.6) | 14,008 (93.7, 93.1-94.2) | 8,797 (87.2, 86.2-88.1) | 1,547 (80.7, 78.2-83.0) |
| Mild (n, %, 95% CI*) | 108 (1.0, 0.8-1.2) | 147 (1.0, 0.8-1.2) | 188 (1.0, 0.9-1.2) | 257 (1.7, 1.4-1.9) | 277 (2.3, 1.9-2.7) | 225 (2.8, 2.3-3.2) | 56 (3.4, 2.4-4.8) |
| Moderate (n, %, 95% CI*) | 25 (0.2, 0.2-0.4) | 43 (0.3, 0.2-0.4) | 93 (0.6, 0.4-0.7) | 193 (0.9, 0.8-1.1) | 570 (3.7, 3.3-4.1) | 856 (8.5, 7.8-9.3) | 273 (12.6, 10.7-14.8) |
| Severe (n, %, 95% CI*) | 1 (0.0, 0.0-0.1) | 0 (0.0) | 5 (0.0, 0.0-0.1) | 13 (0.1, 0.0-0.1) | 60 (0.4, 0.3-0.5) | 148 (1.6, 1.2-2.0) | 72 (3.2, 2.3-4.5) |

*Estimated prevalence and 95% Confidence interval
